# Eye Lens Radiation Dose to Nurses during Cardiac Interventional Radiology: An Initial Study

**DOI:** 10.3390/diagnostics13183003

**Published:** 2023-09-20

**Authors:** Ayumi Yamada, Yoshihiro Haga, Masahiro Sota, Mitsuya Abe, Yuji Kaga, Yohei Inaba, Masatoshi Suzuki, Norio Tada, Masayuki Zuguchi, Koichi Chida

**Affiliations:** 1 Course of Radiological Technology, Health Sciences, Tohoku University Graduate School of Medicine, 2-1 Seiryo, Aoba-ku, Sendai 980-8575, Japan; ayumi.yamada.s4@dc.tohoku.ac.jp (A.Y.); yoshihiro.hg@gmail.com (Y.H.); masahiro.st63728@gmail.com (M.S.); inabay@tohoku.ac.jp (Y.I.); masatoshi.suzuki.c7@tohoku.ac.jp (M.S.); qqrm6wq9k@arrow.ocn.ne.jp (M.Z.); 2 Department of Radiology, Sendai Kousei Hospital, 4-5 Hirose-machi, Aoba-ku, Sendai 980-0873, Japan; mitsuya@sendai-kousei-hospital.jp (M.A.); kaga@sendai-kousei-hospital.jp (Y.K.); 3 Department of Radiation Disaster Medicine, International Research Institute of Disaster Science, Tohoku University, 468-1 Aramaki Aza-Aoba, Aoba-ku, Sendai 980-0845, Japan; 4 Department of Cardiovascular Medicine, Sendai Kousei Hospital, 4-5 Hirose-machi, Aoba-ku, Sendai 980-0873, Japan; noriotada@icloud.com

**Keywords:** radiation protection and safety, interventional radiology (IVR), fluoroscopically guided interventional procedures, percutaneous coronary intervention (PCI), eye lens dose, nurse, occupational radiation exposure, X-ray examination, disaster medicine, resilience

## Abstract

Although interventional radiology (IVR) is preferred over surgical procedures because it is less invasive, it results in increased radiation exposure due to long fluoroscopy times and the need for frequent imaging. Nurses engaged in cardiac IVR receive the highest lens radiation doses among medical workers, after physicians. Hence, it is important to measure the lens exposure of IVR nurses accurately. Very few studies have evaluated IVR nurse lens doses using direct dosimeters. This study was conducted using direct eye dosimeters to determine the occupational eye dose of nurses engaged in cardiac IVR, and to identify simple and accurate methods to evaluate the lens dose received by nurses. Over 6 months, in a catheterization laboratory, we measured the occupational dose to the eyes (3 mm dose equivalent) and neck (0.07 mm dose equivalent) of nurses on the right and left sides. We investigated the relationship between lens and neck doses, and found a significant correlation. Hence, it may be possible to estimate the lens dose from the neck badge dose. We also evaluated the appropriate position (left or right) of eye dosimeters for IVR nurses. Although there was little difference between the mean doses to the right and left eyes, that to the right eye was slightly higher. In addition, we investigated whether it is possible to estimate doses received by IVR nurses from patient dose parameters. There were significant correlations between the measured doses to the neck and lens, and the patient dose parameters (fluoroscopy time and air kerma), implying that these parameters could be used to estimate the lens dose. However, it may be difficult to determine the lens dose of IVR nurses accurately from neck badges or patient dose parameters because of variation in the behaviors of nurses and the procedure type. Therefore, neck doses and patient dose parameters do not correlate well with the radiation eye doses of individual IVR nurses measured by personal eye dosimeters. For IVR nurses with higher eye doses, more accurate measurement of the radiation doses is required. We recommend that a lens dosimeter be worn near the eyes to measure the lens dose to IVR nurses accurately, especially those exposed to relatively high doses.

## 1. Introduction

Interventional radiology (IVR) plays a major role in cardiovascular disease diagnosis and treatment [1,2,3]. IVR is performed using X-ray imaging equipment, catheters, and needles. Although IVR is preferred over surgical procedures because it is less invasive, it results in increased radiation exposure due to long fluoroscopy times and the need for frequent imaging [4,5,6,7,8,9,10,11,12]. Safety measures to prevent radiation exposure are important due to the risk of radiation-induced injuries, such as skin damage in patients and cataracts in medical staff [13,14,15,16,17,18,19,20]. Therefore, increasing attention is being paid to radiation safety and protection for patients and medical staff, particularly related to IVR [21,22,23,24,25,26,27,28].

In 2011, the International Commission on Radiological Protection (ICRP) reduced the recommended threshold dose to prevent radiation-induced cataracts from 8 to 0.5 Gy [29]. Therefore, the ICRP recommended reducing the occupational lens dose limit from 150 to 20 mSv/year over 5 years, with less than 50 mSv in any year [29]. This makes it increasingly important to measure occupational lens doses accurately.

Radiation-induced cataracts have been reported in both IVR physicians and IVR nurses. Therefore, the eye lens doses to IVR nurses must be carefully evaluated. ICRP Publication 85 reported lens opacities in two IVR surgeons and two IVR nurses [30]. In IVR, the highest lens dose reported was for physicians, followed by nurses [31,32]. Wilson-Stewart et al. reported that IVR nurses had higher lens doses than doctors [33]. Hence, it is important to measure the lens exposure of nurses and physicians accurately [34,35,36,37,38,39,40,41]. For this purpose, dosimeters should be worn as close to the eye as possible, and 3 mm dose equivalents should be measured [42].

Although a high dose of scattered radiation reaches the left side of most IVR physicians, this may not be the case for IVR nurses. Therefore, it is important to investigate the left–right differences in lens and neck doses for IVR nurses. The purpose of our study was to determine the occupational eye dose of cardiac IVR nurses using a direct eye dosimeter, and investigate simple methods to manage their lens dose. Over 6 months, in a catheterization laboratory, we measured the occupational dose to the eyes and neck of nurses on the right and left sides.

## 2. Materials and Methods

### 2.1. Subjects

This study included 1065 cardiovascular procedures performed over a 6-month period between October 2017 and March 2018 at Sendai Kosei Hospital, Japan (Table 1). However, for various reasons, the occupational exposures of one and two nurses were measured over 5- and 4-month periods, respectively. Although these periods were somewhat shorter (spanning 5 or 4 months), we believe that they had negligible impact on the study outcomes. These procedures included a variety of therapeutic and diagnostic IVR procedures, including percutaneous coronary intervention (PCI), catheter ablation (ABL), and pacemaker implantation (PMI).

The occupational exposure of six catheterization laboratory nurses was measured using a dosimeter at 1-month intervals within the study period. During the cardiovascular procedures, the nurses wore protective aprons (usually composed of a 0.35 mm Pb equivalent) but not Pb glasses. We used an under-the-table X-ray fluoroscopy system with a flat panel detector. The control system for this equipment automatically sets the X-ray output. Digital cine acquisition was performed at 15 frames per second for the PCI procedures. Patient dose parameters (fluoroscopy time [FT] and air kerma [AK]) were recorded on the system for each procedure and analyzed monthly.

**Figure 1:**
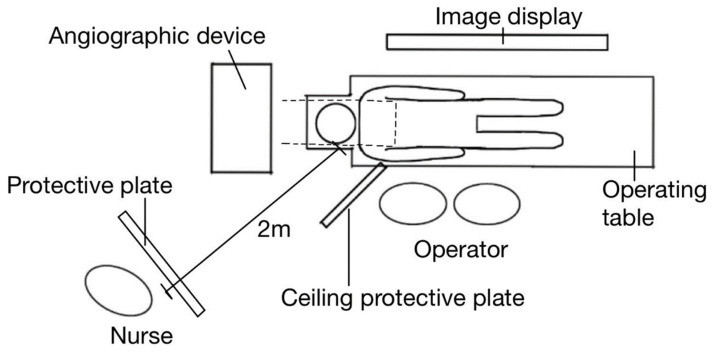
Positions of equipment and medical workers. IVR nurses were positioned 2 m above the patient on the right side, but the position varied during the procedure.

The nurses were positioned approximately 2 m above the right side of the patient (Figure 1), but changed position frequently during the procedures.

### 2.2. Dosimetry

**Figure 2:**
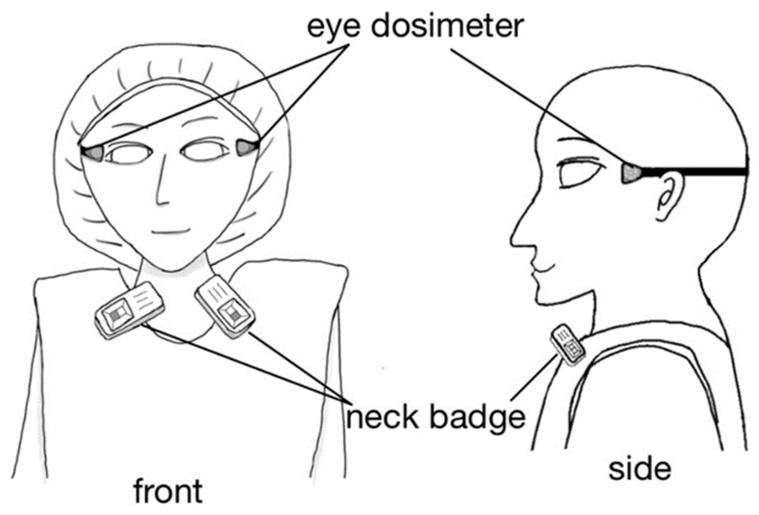
IVR-nurse dosimeter positions.

We measured the lens radiation dose of nurses using the direct eye lens dosimeter DOSIRIS™ (IRSN, Croisy-sur-Seine, France) (Figure 2), which measures 3 mm dose equivalents [H_p_(3)] to the eye lens. The dosimeter has excellent measurement performance in the diagnostic X-ray energy range [43]. DOSIRIS™ uses a thermoluminescent dosimeter (TLD). The detector is a TLD (^7^LiF:Mg,Ti) integrated in a 3 mm thick polypropylene cap. Chiyoda Technol Corporation (Tokyo, Japan) supplied and calibrated the lens dosimeters, and calculated the cumulative dose over 1 month. The nurses wore the dosimeter close to both eyes during the monitoring period.

We also estimated the lens dose using a silver-activated phosphate glass personal dosimeter (Figure 2) that measures 0.07 mm dose equivalents ([H_p_(0.07)]; Glass Badge, Chiyoda Technol Corp.). The neck badge (“Glass Badge”) dosimeter measures the H_p_(0.07) and 10 mm dose equivalents [H_p_(10)]. However, the neck badge cannot measure the H_p_(3). In Japan, eye doses are estimated using the larger of the H_p_(0.07) and H_p_(10) neck badge doses. In this study, we employed the H_p_(0.07) values; these are at least as high as the H_p_(10) values.

The badge was worn on the outside of the lead apron on the left side and on the right side of the neck. We evaluated the correlation between lens dosimeter [H_p_(3)] and neck badge [H_p_(0.07)] doses to the left and right sides, respectively, to determine whether the lens dose assessed using the neck badge was accurate. We also compared the left- and right-side doses using the lens dosimeter and neck badge, and determined the appropriate position for the dosimeter. Furthermore, we investigated whether exposure doses to nurses can be estimated from the patient dose parameters of total FT and AK. In our institute, FT and AK data are recorded routinely.

### 2.3. Statistical Analysis

We used JMP Pro (version 15.0; Cary, NC, USA) for the statistical analysis. We leveraged the known dataset to calculate the coefficient of determination (R^2^) for validation, confirming that consistent results were obtained. The Wilcoxon signed-rank test was used to compare the two groups. A *p* value < 0.05 was considered statistically significant. The correlations between lens dosimeter and neck badge measurements are presented as 95% confidence intervals. We used Microsoft Excel to determine the correlation coefficient (*r*) of neck and lens doses with patient dose parameters. Averages presented in tables are calculated using all data sets. 

## 3. Results

Table 2 presents the average monthly lens dosimeter and neck badge doses of the nurses. The nurse (E) with the highest dose (DOSIRIS, left and right eye = 0.74 and 0.70 mSv/month; neck badges, left and right = 1.10 and 1.28 mSv/month, respectively) also assisted in the highest number of procedures (total = 424; Table 1). Table 3 shows the IVR nurse radiation dose per procedure.

### 3.1. Correlation and Comparison of Lens Dosimetry and Neck Badge

**Figure 3:**
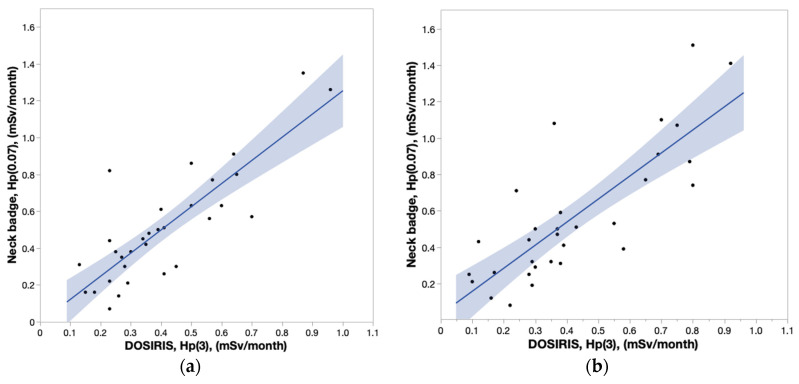
(**a**) Correlation coefficient (95% confidence interval) between DOSIRIS and neck badge doses to IVR nurses on the left side. R^2^ = 0.6968. (**b**) Correlation coefficient (95% confidence interval) between DOSIRIS and neck badge doses to IVR nurses on the right side. R^2^ = 0.6508.

**Figure 4:**
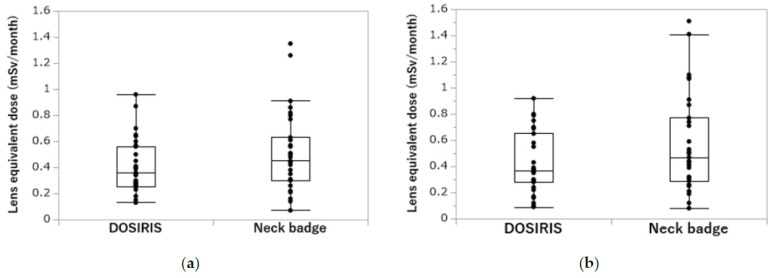
(**a**) Comparison of IVR nurse neck badge and DOSIRIS doses on the left side. (*p* = 0.2368). (**b**) Comparison of IVR nurse neck badge and DOSIRIS doses on the right side. (*p* = 0. 1393).

Figure 3a,b depict the correlation between DOSIRIS and neck badge measurements. The coefficients of determination (R^2^) for the left eye and left neck, and for the right eye and right neck were 0.6968 and 0.6508, respectively, indicating significant correlations. The lens and neck badge doses were not significantly different (left, *p* = 0.2368; right, *p* = 0.1393), but the dose measured by the neck badge tended to be larger than that measured by the dosimeter (Figure 4a,b).

### 3.2. Left–Right Difference in Lens and Neck Doses

**Figure 5:**
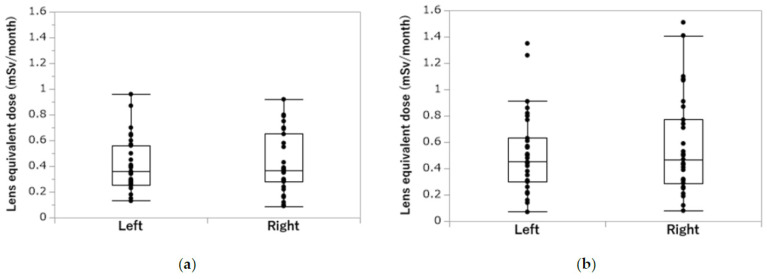
(**a**) Left–right difference in IVR nurse DOSIRIS doses. (*p* = 0. 8769). (**b**) Left–right difference in IVR nurse neck badge doses. (*p* = 0. 7038).

Figure 5a,b show that there were no significant left–right differences in the lens and neck doses, respectively (lens dose, *p* = 0.8769; neck badge, *p* = 0.7038). There was only a minor difference between the left and right sides, with the dose being slightly higher on the right side than on the left side in both the DOSIRIS and neck badge measurements.

### 3.3. Correlation of Lens and Neck Doses with Patient Dose Parameters

**Figure 6:**
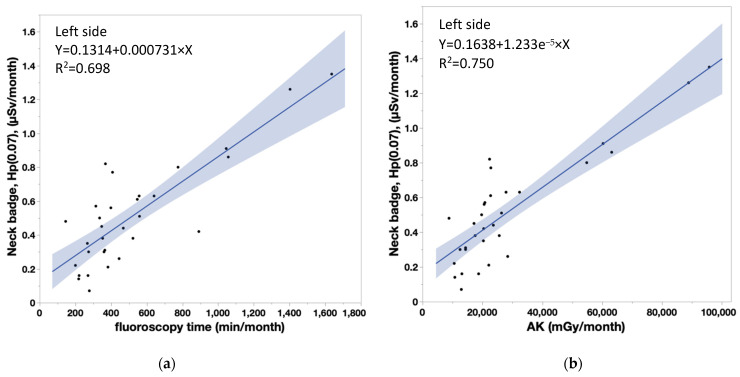
(**a**) Correlation coefficient (95% confidence interval) between the patient dose parameter (FT) and neck badge doses to IVR nurses on the left side. Y = 0.1314 + 0.000731 × X, R^2^ = 0.698. (**b**) Correlation coefficient (95% confidence interval) between the patient dose parameter (AK) and neck badge doses to IVR nurses on the left side. Y = 0.1638 + 1.233 × 10^−5^ × X, R^2^ = 0.750.

**Figure 7:**
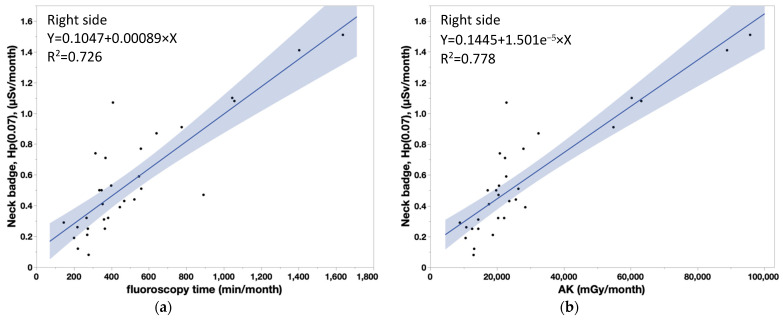
(**a**) Correlation coefficient (95% confidence interval) between the patient dose parameter (FT) and neck badge doses to IVR nurses on the right side. Y = 0.1047 + 0.00089 × X, R^2^ = 0.726. (**b**) Correlation coefficient (95% confidence interval) between the patient dose parameter (AK) and neck badge doses to IVR nurses on the right side. AK: Y = 0.1445 + 1.501 × 10^−5^ × X, R^2^ = 0.778.

**Figure 8:**
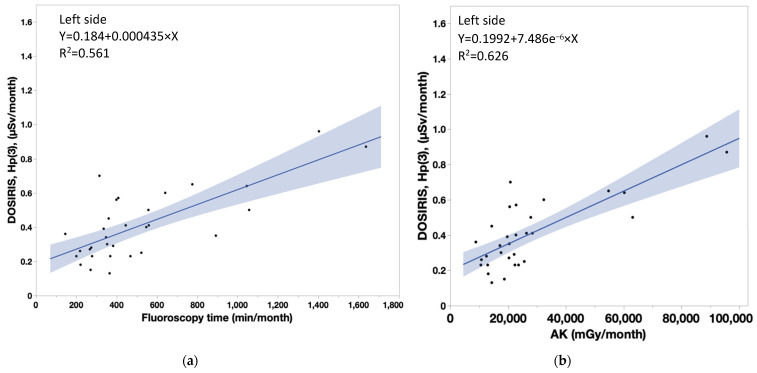
(**a**) Correlation coefficient (95% confidence interval) between the patient dose parameter (FT) and the DOSIRIS measurements to IVR nurses on the left side. Y = 0.184 + 0.000435 × X, R^2^ = 0.561. (**b**) Correlation coefficient (95% confidence interval) between the patient dose parameter (AK) and the DOSIRIS measurements to IVR nurses on the left side. Y = 0.1992 + 7.486 × 10^−6^ × X, R^2^ = 0.626.

**Figure 9:**
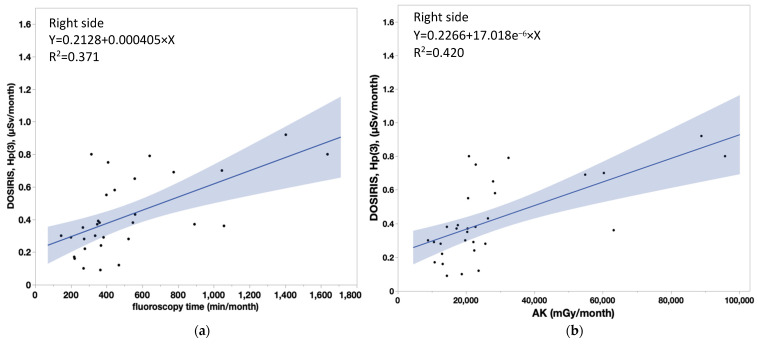
(**a**) Correlation coefficient (95% confidence interval) between the patient dose parameter (FT) and the DOSIRIS measurements to IVR nurses on the right side. Y = 0.2128 + 0.000405 × X, R^2^ = 0.371. (**b**) Correlation coefficient (95% confidence interval) between the patient dose parameter (AK) and the DOSIRIS measurements to IVR nurses on the right side. AK: Y = 0.2256 + 7.018 × 10^−6^ × X, R^2^ = 0.420.

Table 4 shows that the coefficient of determination (R^2^) between measured doses in nurses and patient dose parameters were significant (neck doses, *r* > 0.8 for all). Similarly, there were significant correlations between lens doses and patient dose parameters, although the correlation coefficients were smaller for the lens dose than the neck dose. Figure 6a,b show the correlations between the neck badge measurements on the left side and the patient dose parameters (FT: Y = 0.1314 + 0.000731 × X, AK: Y = 0.1638 + 1.233 × 10^−5^ × X). Figure 7a,b show the correlations between the neck badge measurements on the right side and the patient dose parameters (FT: Y = 0.1047 + 0.00089 × X, AK: Y = 0.1445 + 1.501 × 10^−5^ × X). Figure 8a,b show the correlations between the DOSIRIS measurements on the left side and the patient dose parameters (FT: Y = 0.184 + 0.000435 × X, AK: Y = 0.1992 + 7.486 × 10^−6^ × X). Figure 9a,b show the correlations between the neck badge measurements on the right side and the patient dose parameters (FT: Y = 0.2128 + 0.000405 × X, AK: Y = 0.2256 + 7.018 × 10^−6^ × X).

## 4. Discussion

Very few studies have evaluated IVR nurse lens doses using direct dosimeters. Here, we focused on these nurses and used DOSIRIS to obtain eye dose data.

Currently, lens doses are generally assessed using personal dosimeters worn on the chest or neck [44,45]. In this study, we investigated whether the neck badge dose accurately represents the lens dose. Dosimeters were worn on the left and right sides of the lens and neck, and we examined the appropriate position for the dosimeter. Few studies have estimated exposure doses to nurses based on patient dose parameters [36,46], although some studies have been conducted among doctors [47,48,49]. Therefore, we investigated whether the exposure doses of nurses can be estimated from the patient dose parameters of FT and AK.

To the best of our knowledge, this study was the first to use direct lens dosimetry to measure left–right differences in the lens doses received by nurses engaged in cardiac IVR over a period of 6 months.

### 4.1. Comparison of Lens Dosimetry and Neck Badge Doses

If a neck badge (conventional method) can accurately measure the eye dose for IVR nurses, the use of an additional direct eye dosimeter is unnecessary; consequently, there is no additional cost. We examined whether it is appropriate to estimate the lens dose from the neck badge dose, and found a significant correlation between the two. Therefore, it may be possible to estimate the lens dose from neck badge dose. However, many correlation values were outside the 95% confidence interval, which implies that the results vary between nurses and procedures. Therefore, we recommend the use of a direct lens dosimeter to measure the lens dose of IVR nurses accurately. Our results also imply that neck badges may overestimate the lens doses to cardiac IVR nurses, as evidenced by the mean value of the neck badge dose/lens dose being greater than 1 on both sides. This may have been because nurses are often close to the patient during ABL, which exposes their neck to the scattered radiation [50,51,52,53,54,55,56,57,58]. In line with our results, a previous study reported that neck badges may overestimate the lens dose of nurses during interventional cardiology procedures [31]. 

The coefficient of determination for lens and neck doses to cardiac IVR doctors on the left side without the use of protective glasses in that study was 0.8593 [31], which was greater than that to nurses in our study (left side = 0.6968, right side = 0.6508).

Although the lens dose was previously reported to be higher than the neck dose during respiratory endoscopy using X-ray fluoroscopy [59], this was probably due to the use of an over-the-table X-ray fluoroscopy system.

In our study, nurse E had the highest exposure dose, which was due to nurse E being present for the highest number of procedures. Therefore, nurse E had the highest annual dose (average monthly dose × 12; DOSIRIS, left and right eye = 8.9 and 8.4 mSv/y; neck badges, left and right = 13.2 and 15.4 mSv/y, respectively), although it did not exceed the annual dose limit recommended by ICRP (20 mSv/y over 5 years). In cases with a high eye dose (such as nurse E for whom the dose limit would be surpassed with an addition of ~5 mSv), additional lens dosimeters should be worn near the eyes to accurately measure the lens dose of IVR nurses. To protect IVR nurses, the ocular radiation eye dose needs to be determined with greater precision, especially that of those who receive high doses.

Importantly, nurse E also had the lowest dose per procedure, probably because nurse E was not involved in ABL, which leads to exposure to relatively high-radiation doses. Conversely, nurse B had the highest dose per procedure, which may have been due to improper use of the protective plate and maintaining a position close to the patient. Hence, awareness of the appropriate use of protective devices during procedures is necessary. Feedback on these findings will help to improve nursing practices.

### 4.2. Left-Right Differences in Lens and Neck Doses

In general, IVR physicians have a higher radiation dose on the left than right side, because it is nearer to the source of scattered radiation [60,61,62,63,64,65]; therefore, IVR physicians wear the lens dosimeter near the left eye.

Our study is the first to evaluate the left–right differences in lens doses measured directly using an eye dosimeter, and neck doses to nurses engaged in cardiac IVR. The lens dose was slightly higher on the right side than on the left side; therefore, it may be preferable to wear the lens dosimeter near the right eye. However, this needs to be confirmed by further studies.

### 4.3. Correlations of Lens and Neck Doses with Patient Dose Parameters

Although Principi et al. previously reported the correlation between lens doses to IVR nurses and dose area product (DAP) (R^2^ = 0.5–0.6) [46], no studies have investigated the correlation between lens doses to nurses and FT or AK. Therefore, we investigated whether the lens dose to nurses can be evaluated from the patient dose parameters of FT and AK.

We found that neck and lens doses had significant correlations (*r* ≥ 0.8 and <0.8, respectively) with patient dose parameters (FT and AK), although the correlation coefficients were smaller for the eye dose than for the neck dose. In essence, reducing the patient dose decreases IVR nurse exposure, while increasing the patient dose escalates IVR nurse exposure.

Accurate assessment of the lens dose to nurses based on patient dose parameters may be difficult. In addition, the lens dose will vary depending on nurses’ habits and the procedure. Therefore, neck doses and patient dose parameters do not correlate well with the radiation eye doses of individual IVR nurses measured by personal eye dosimeters. Hence, we recommend the use of a direct lens dosimeter to measure the lens dose to nurses accurately. 

In summary, nurses engaged in cardiac IVR receive the highest lens radiation doses among medical workers, after physicians. Hence it is important to measure the lens exposure of IVR nurses accurately. We used a direct dosimeter that measures 3 mm dose equivalents to measure the occupational exposure dose of nurses; we calculated the eye and neck doses on the right and left sides. There was little difference in eye doses between the right and left sides (lens dose, *p* = 0.8769; neck dose, *p* = 0.7038), but the mean value was slightly higher in the right eye. Therefore, if used, the dosimeter should be worn near the right eye. In addition, there were significant correlations between the measured dose and patient dose parameters (FT and AK), which may allow for the estimation of the lens dose from the patient dose. However, it is difficult to measure the lens dose accurately using patient dose parameters because of variation in the behavior of nurses and the procedure type. We recommend the use of direct lens dosimeters to measure the lens dose received by IVR nurses accurately.

## 5. Conclusions

We performed an initial study of occupational eye exposure for IVR nurses. We measured the lens and neck doses of cardiac IVR nurses, as well as their correlations. In addition, we determined the appropriate position for dosimeters. There was a significant correlation between lens and neck doses, although many correlation values were outside the 95% confidence interval. Therefore, it may be possible to estimate the lens dose from a neck badge. In addition, we investigated whether it is possible to estimate the dose of nurses from patient dose parameters, and found significant correlations between the measured dose (neck and lens doses) and patient dose parameters.

However, it may be difficult to determine the lens dose to nurses accurately from neck badge and patient dose parameters because of variation in the behavior of nurses and procedure types. Therefore, neck doses and patient dose parameters do not correlate well with the radiation eye doses of individual IVR nurses measured by personal eye dosimeters.

For IVR nurses with higher eye doses, more accurate measurement of the radiation doses is required. Therefore, they should wear lens dosimeters near the eyes to measure the lens dose accurately, especially those exposed to relatively high doses.

## Figures and Tables

**Table 1:** General characteristics of procedures for IVR nurses (mean ± SD).

	Total Number of Procedures (Treatment (PCI: ABL: Others): Diagnosis)	Average Number of Procedures Per Month	Fluoroscopy Time Per Month (min.)	Air Kerma Per Month (mGy)
Nurse A	89 [59 (22:19:18):30]	14.8 ± 5.0	270.5 ± 63.3	15266 ± 4731
Nurse B **	79 [48 (18:15:15):31]	13.8 ± 7.6	370.5 ± 42.6	19637 ± 3658
Nurse C	196 [98 (42:26:30):98]	32.7 ± 11.7	575.4 ± 218.4	27190 ± 13828
Nurse D *	170 [95 (39:28:28):75]	35.3 ± 9.5	511.1 ± 112.6	26528 ± 5484
Nurse E **	424 [158 (136:0:22):266]	106.0 ± 19.8	1285.4 ± 286.8	77024 ± 17916
Nurse F	108 [63 (23:20:20):45]	18.0 ± 4.4	300.7 ± 114.3	16003 ± 5672

Measurement period: * 5 months; ** 4 months.

**Table 2:** Monthly dose of IVR nurses (mean ± SD).

	DOSIRIS, H_p_(3), (mSv/Month)		Neck Badge, H_p_(0.07), (mSv/Month)		Neck Badge/DOSIRIS	
	Left	Right	Left	Right	Left	Right
Nurse A	0.25 ± 0.04	0.27 ± 0.07	0.22 ± 0.10	0.21 ± 0.10	0.87 ± 0.34	0.78 ± 0.25
Nurse B	0.57 ± 0.10	0.62 ± 0.19	0.55 ± 0.19	0.66 ± 0.32	0.95 ± 0.30	1.03 ± 0.27
Nurse C	0.37 ± 0.15	0.39 ± 0.16	0.58 ± 0.19	0.60 ± 0.18	1.72 ± 0.91	1.67 ± 0.64
Nurse D	0.44 ± 0.11	0.57 ± 0.16	0.48 ± 0.16	0.59 ± 0.22	1.10 ± 0.22	1.04 ± 0.21
Nurse E	0.74 ± 0.21	0.70 ± 0.24	1.10 ± 0.27	1.28 ± 0.22	1.50 ± 0.18	2.00 ± 0.69
Nurse F	0.25 ± 0.11	0.18 ± 0.10	0.34 ± 0.16	0.32 ± 0.11	1.42 ± 0.65	2.10 ± 0.94
Average	0.39 ± 0.12	0.40 ± 0.15	0.53 ± 0.27	0.58 ± 0.35	1.26 ± 0.34	1.44 ± 0.56

**Table 3:** Dose of IVR nurses per procedure (mSv/procedure).

	DOSIRIS, H_p_(3), (mSv/Procedure)		Neck Badge, H_p_(0.07), (mSv/Procedure)	
	Left	Right	Left	Right
Nurse A	0.0167	0.0179	0.0147	0.0144
Nurse B	0.0292	0.0318	0.0293	0.0340
Nurse C	0.0113	0.0119	0.0178	0.0185
Nurse D	0.0131	0.0167	0.0142	0.0174
Nurse E	0.0070	0.0066	0.0103	0.0120
Nurse F	0.0141	0.0100	0.0188	0.0180
Average	0.0152 ± 0.0076	0.0158 ± 0.0089	0.0175 ± 0.0065	0.0189 ± 0.0077

**Table 4:** Coefficient of determination (R^2^) of IVR nurse neck and lens doses with patient dose parameters.

	Fluoroscopy Time Per Month	Air Kerma Per Month
Neck dose		
Left	0.698	0.750
Right	0.726	0.778
Lens dose		
Left	0.561	0.626
Right	0.371	0.420

## Data Availability

Not applicable.

## Abbreviations

IVR	interventional radiology
PCI	percutaneous coronary intervention
PMI	pacemaker implantation
AK	air kerma
ICRP	International Commission on Radiological Protection
ABL	catheter ablation
FT	fluoroscopy time
